# Restoration and Enhancement of Historical Stereo Photos †

**DOI:** 10.3390/jimaging7070103

**Published:** 2021-06-24

**Authors:** Marco Fanfani, Carlo Colombo, Fabio Bellavia

**Affiliations:** 1 Department of Information Engineering, Università degli Studi di Firenze, 50139 Firenze, Italy; marco.fanfani@unifi.it (M.F.); carlo.colombo@unifi.it (C.C.); 2 Department of Math and Computer Science, Università degli Studi di Palermo, 90123 Palermo, Italy

**Keywords:** image denoising, image restoration, image enhancement, stereo matching, optical flow, gradient filtering, stacked median, guided supersampling, historical photos

## Abstract

Restoration of digital visual media acquired from repositories of historical photographic and cinematographic material is of key importance for the preservation, study and transmission of the legacy of past cultures to the coming generations. In this paper, a fully automatic approach to the digital restoration of historical stereo photographs is proposed, referred to as Stacked Median Restoration plus (SMR+). The approach exploits the content redundancy in stereo pairs for detecting and fixing scratches, dust, dirt spots and many other defects in the original images, as well as improving contrast and illumination. This is done by estimating the optical flow between the images, and using it to register one view onto the other both geometrically and photometrically. Restoration is then accomplished in three steps: (1) image fusion according to the stacked median operator, (2) low-resolution detail enhancement by guided supersampling, and (3) iterative visual consistency checking and refinement. Each step implements an original algorithm specifically designed for this work. The restored image is fully consistent with the original content, thus improving over the methods based on image hallucination. Comparative results on three different datasets of historical stereograms show the effectiveness of the proposed approach, and its superiority over single-image denoising and super-resolution methods. Results also show that the performance of the state-of-the-art single-image deep restoration network Bringing Old Photo Back to Life (BOPBtL) can be strongly improved when the input image is pre-processed by SMR+.

## 1. Introduction

Photographic material of the XIX and XX centuries is an invaluable source of information for historians of art, architecture and sociology, as it allows them to track the changes occurred over the decades to a community and its living environment. Unfortunately, due to the effect of time and bad preservation conditions, most of the survived photographic heritage is partially damaged, and needs restoration, both at the physical (cardboard support, glass negatives, films, etc.) and digital (the image content acquired through scanners) levels. Dirt, scratches, discoloration and other signs of aging strongly reduce the visual quality of photos [1]. A similar situation also holds for the cinematographic material [2].

**Figure 1:**
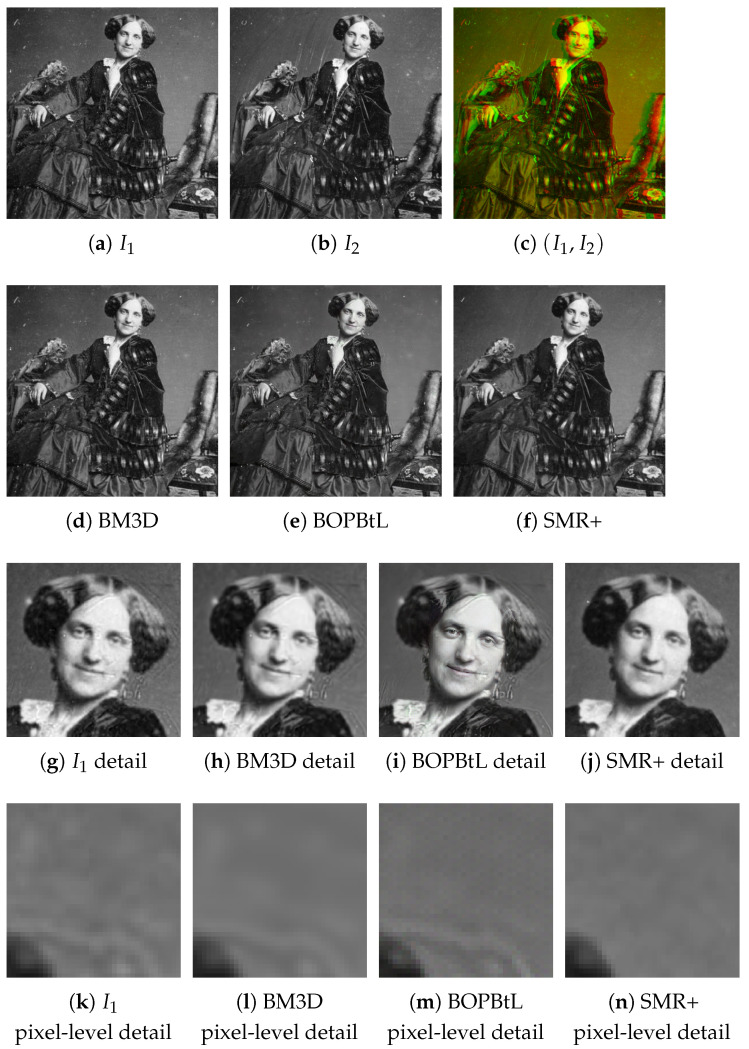
First row: An example of historical stereo pair of images, $I_{1}$ and $I_{2}$, also superimposed as anaglyph. Second row: Enhancement of $I_{1}$ according to different methods, including the proposed SMR+ method. Although visually impressive, the deep super-resolution result of BOPBtL does not preserve the true input image. Third row: A detail of $I_{1}$ and the restored images according to the different methods. A closer look at BOPBtL reveals alterations with respect to the original face expression, accentuating the smile and introducing bush-like textures on the hair. Fourth row: pixel level detail of $I_{1}$ and of the restored images according to the different methods. The specific image region considered is the background around the right shoulder. Notice the chessboard-like texture pattern typical of the deep network approaches, not visible at coarser scales. Best viewed in color: the reader is invited to zoom in on the electronic version of the manuscript in order to better appreciate the visual differences.

Digital restoration of both still images and videos has attracted considerable interest from the research community in the early 2000s. This has led to the development of several tools that improve the visual quality. Some approaches rely on the instantiation of noise models, which can either be fixed a priori or derived from the input images [3,4,5]. Other approaches detect damaged areas of the image and correct them according to inpainting techniques [6]. Self-correlation inside the image, or across different frames in videos, is often exploited in this context, under the assumption that zero-mean additive noise cancels out as the available number of image data samples increases [7,8,9]. A similar idea is exploited by super-resolution techniques that enhance image quality by pixel interpolation [10,11]. In recent years, the algorithmic methods above have been sided by methods based on deep learning that can infer the image formation model or the scene content [12] from a training set in order to inject this information into the final output, a process called image hallucination [13,14,15]. Although the final image may often alter the original image data content, and hence cannot be fully trusted (e.g., in the medical diagnosis domain), the hallucination methods can give visually pleasing results (see Figure 1).

Stereoscopy has accompanied photography since its very birth in the nineteenth century, with ups and downs in popularity through time. Notwithstanding the lesser spread of stereo photography with respect to standard (monocular) photography, many digital archives with thousands of stereo images exist, some of which are freely available on the web. Stereo photos have a richer content than standard ones, as they present two different views of the same scene, thus explicitly introducing content redundancy and implicitly embedding information about scene depth. This characteristic can be exploited also in digital noise removal, enhancement and restoration, since a damaged area in one image can be reconstructed from the other image, provided that the correspondences between the two images are known. At a first glance, the above-mentioned approach looks similar to that of video restoration from multiple video frames, in which the scene is acquired in subsequent time instants from slightly changed viewpoints. However, stereo images have their own peculiarities, and actually introduce in the restoration process more complications than video frames, which in movies typically exhibit an almost static and undeformed background, differently from stereo pairs. As a matter of fact, although several advances have been recently made in stereo matching and dense optical flow estimation [16], the problem is hard and far from being fully solved, especially in the case of very noisy and altered images such as those generated by early photographic stereo material. To the best of the authors’ knowledge, stereo photo characteristics have been employed only for the super-resolution enhancement or deblurring of modern, clean photos [17,18,19]. On the other hand, the image analysis and computer vision approaches developed so far for historical stereo photos mainly aimed at achieving (usually in a manual way) better visualizations or 3D scene reconstructions [20,21,22], with no attempt at restoring the quality of the raw stereo pairs.

This paper proposes a new approach to clean up and restore the true scene content in degraded historical stereo photographs, named Stacked Median Restoration Plus (SMR+), extending our previous work [23], and working in a fully automatic way. With respect to existing single image methods, damaged image areas with scratches or dust can be better detected and fixed, thanks to the availability of more sampled data points for denoising. In addition, the correct illumination can be restored or enhanced in a way akin to that of High Dynamic Range Imaging, where the images of the same scene taken at different exposure levels are used in order to enhance details and colors [24]. For this scope, the optical flow, estimated with the recent state-of-the-art Recurrent All-Pairs Field Transforms (RAFT) deep network [16], is used to synthesize corresponding scene viewpoints in the stereo pair, while denoising and restoration are carried out using novel yet non-deep image processing approaches. The entire process is superseded by scene content consistency validation, used to check critical stereo matching mispredictions that were left unresolved by the network. Our approach aims to obtain an output which is fully consistent with the original scenario captured by the stereo pair, in contrast with the recent super-resolution and denoising approaches based on image hallucination.

This paper extends our previous work [23], hereafter reported as Stacked Median Restoration (SMR) under several aspects:

- With respect to SMR, the novel SMR+ is redesigned so as to better preserve finer details while at the same time improving further the restoration quality. This is accomplished by employing supersampling [25] at the image fusion step in conjunction with a weighting scheme guided by the original restoration approach.
- The recent state-of-the-art deep network BOPBtL [26], specifically designed for old photo restoration, is now included in the comparison, both as standalone and to serve as post-processing of SMR+.
- The collection of historical stereo photos employed as a dataset is roughly doubled to provide a more comprehensive evaluation.
- The use of renowned image quality assessment metrics is investigated and discussed for these kinds of applications.

The rest of the paper is organized as follows: Section 2 introduces the proposed approach. An experimental evaluation and comparison with similar approaches is reported in Section 3. Finally, conclusions and future work are discussed in Section 4.

*Note: To ease the inspection and the comparison of the different images presented, an interactive PDF document is provided in the additional material (https://drive.google.com/drive/folders/1Fmsm50bMMDSd0z4JXOhCZ3hPDIXdwMUL) to allow readers to view each image at its full dimensions and quickly switch to the other images to be compared.*

## 2. Proposed Method

**Figure 2:**
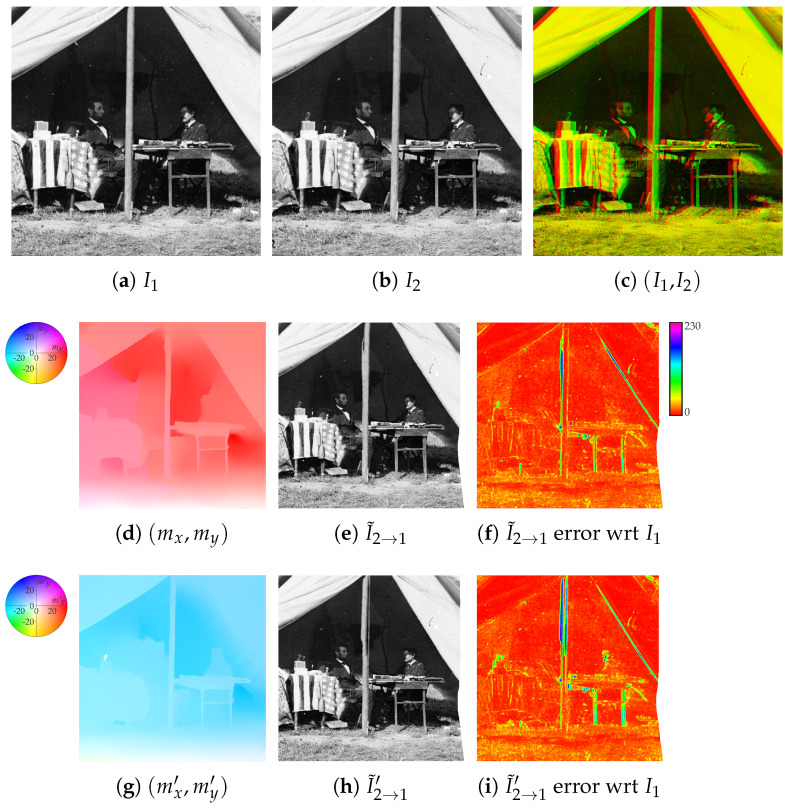

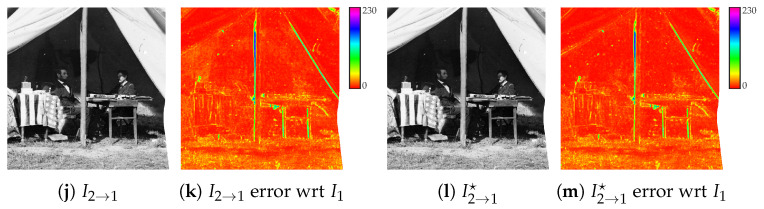
Auxiliary image pointwise transfer and color correction steps (see Section 2.1 and Section 2.2): (**a**) Reference image $I_{1}$, (**b**) auxiliary image $I_{2}$, (**c**) superimposition of $I_{1}$ and $I_{2}$ as anaglyph, (**d**) visual representation of the optical flow map $\left(m_{x},m_{y}\right)$ extracted by RAFT, (**e**) image ${\tilde{I}}_{2\to 1}$ as resynthesis of $I_{2}$ through $\left(m_{x},m_{y}\right)$ and (**f**) its error with respect to $I_{1}$, (**g**) visual representation of the optical flow map $\left(m_{x}^{\prime },m_{y}^{\prime }\right)$ extracted by RAFT after switching the input images, (**h**) image ${\tilde{I}}_{2\to 1}$ as resynthesis from $I_{2}$ through $-(m_{x}^{\prime },m_{y}^{\prime })$ and (**i**) its error with respect to $I_{1}$, (**j**) final resynthesized image $I_{2\to 1}$ considering the locally best optical flow estimation between ${\tilde{I}}_{2\to 1}\left(x,y\right)$ and ${\tilde{I}}_{2\to 1}^{\prime }\left(x,y\right)$ and (**k**) its error with respect to $I_{1}$, (**l**) image $I_{2\to 1}^{⋆}$ obtained after applying GPS/LCP color correction to $I_{2\to 1}$ using $I_{1}$ as reference, and (**m**) the corresponding error map with respect to $I_{1}$. Best viewed in color. The reader is invited to zoom into the electronic version of the manuscript in order to better appreciate the visual differences.

Given a pair of stereo images $I_{1}$ and $I_{2}$, the aim of the process is to output a defect-free version of one image of the pair (referred to as the reference) by exploiting the additional information coming from the other image (denoted as auxiliary). For convenience, the reference is denoted as $I_{1}$ (see Figure 2a) and the auxiliary image as $I_{2}$ (see Figure 2b), but their roles can be interchanged. Images are assumed to be single channel graylevel, i.e., $I_{1},I_{2}:R^{2}\to \left[0,255\right]$.

### 2.1. Auxiliary Image Pointwise Transfer

As a first step, the recent state-of-the-art RAFT deep network [16] is used to compute the optical flow map pair $f_{\mathrm{RAFT}}\left(I_{1},I_{2}\right)=\left(m_{x},m_{y}\right)$, where $m_{x},m_{y}:R^{2}\to R$ (see Figure 2d), so that a synthesized image based on the content of $I_{2}$ and registered onto $I_{1}$ can be obtained as

(1) $${\tilde{I}}_{2\to 1}\left(x,y\right)=I_{2}\left(x+m_{x}\left(x,y\right),y+m_{y}\left(x,y\right)\right)$$

by transferring pixel intensity values from $I_{2}$ into the view given by $I_{1}$ (see Figure 2e). Note that spots of missing data can be present on ${\tilde{I}}_{2\to 1}$ when no pixel in $I_{2}$ maps onto the specific image area, due, for instance, to image occlusions. In the error free ideal case, it must hold that $I_{1}={\tilde{I}}_{2\to 1}$ for every correspondence between $I_{1}$ and $I_{2}$. However, in real situations, this may not happen, as shown in Figure 2f reporting the average absolute error between $I_{1}$ and ${\tilde{I}}_{2\to 1}$ on 5×5 local window patches.

Notice also that, in the case of perfectly rectified stereo images, it holds everywhere that $m_{y}\left(x,y\right)=0$. Under this particular setup, in which $m_{x}$ is denoted as *disparity map* and is the only map that needs to be estimated, several classical methods have proven to provide good results while being computationally efficient [27]. However, according to our experience [21], these methods are not feasible in the case of degraded historical stereo photos. First, image degradation due to aging and the intrinsic image noise due to the technological limitations of the period decrease the ability of these methods to find the right correspondences. Second, the output of these methods is quite sensitive to the initial configuration of the parameters and, by considering the variability of the historical acquisition setups, each stereo pair would require the human supervision to get even a sub-optimal result. Third, the stereo alignment for the photos under consideration is far from perfect due to the technological limitations of the period, hence both the maps $m_{x}$ and $m_{y}$ are to be considered. Hence, our choice fell under the state-of-the-art RAFT that provides a sufficiently good initial estimation of the optical flow maps in most cases.

**Figure 3:**
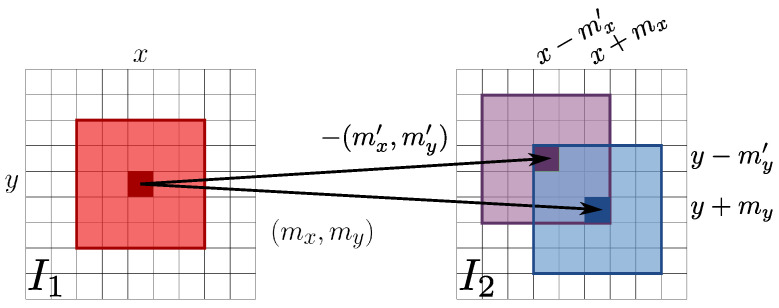
Illustration of the $I_{2\to 1}$ image formation process from the two resynthesized images ${\tilde{I}}_{2\to 1}\left(x,y\right)$ and ${\tilde{I}}_{2\to 1}^{\prime }\left(x,y\right)$, respectively driven by the optical flow estimation maps $\left(m_{x},m_{y}\right)$ and $-(m_{x}^{\prime },m_{y}^{\prime })$. A point (x,y) in $I_{1}$ can be mapped back to $I_{2}$ according to either Equation (1) or Equation (2). The best back-mapping minimizing locally the error among the two possible optical flow estimates is then chosen to form $I_{2\to 1}$. Best viewed in color.

A further flow mapping pair $f_{\mathrm{RAFT}}\left(I_{2},I_{1}\right)=\left(m_{x}^{\prime },m_{y}^{\prime }\right)$ (see Figure 2g) can be obtained by switching the two input images, which can be employed to synthesize a second image according to

(2) $${\tilde{I}}_{2\to 1}^{\prime }\left(x,y\right)=I_{2}\left(x-m_{x}^{\prime }\left(x,y\right),y-m_{y}^{\prime }\left(x,y\right)\right)$$

(see Figure 2h) so that, in the error free ideal case for every correspondence between $I_{1}$ and $I_{2}$, it holds that $\left(m_{x},m_{y}\right)=-\left(m_{x}^{\prime },m_{y}^{\prime }\right)$, which implies that $I_{1}={\tilde{I}}_{2\to 1}={\tilde{I}}_{2\to 1}^{\prime }$. This usually does not happen, as shown by the relative error image of Figure 2i. Indeed, comparing the first and second rows of Figure 2, RAFT optical flow estimation is not completely accurate and does not preserve map inversion when exchanging the input image order. The final synthesized image $I_{2\to 1}$ (see Figure 2j) is then obtained by choosing the intensity value at each pixel (x,y) as the one from ${\tilde{I}}_{2\to 1}\left(x,y\right)$ and ${\tilde{I}}_{2\to 1}^{\prime }\left(x,y\right)$ that minimizes the sum of absolute errors with respect to $I_{1}$ on a small 5×5 local window centered on the pixel (see Figure 3). A smaller error between the final resynthesized image $I_{2\to 1}$ and the reference image $I_{1}$ is obtained (see Figure 2k) with respect to the errors given by ${\tilde{I}}_{2\to 1}\left(x,y\right)$ and ${\tilde{I}}_{2\to 1}^{\prime }\left(x,y\right)$.

### 2.2. Color Correction

Due to the technical limitations of the old photographic instrumentation, illumination conditions between the two stereo images can differ noticeably. For instance, flash lamp and, even more, flash powder did not provide each time uniform and identical illumination conditions, and it was not infrequent that a single camera was moved in two different positions in order to simulate a stereo setup instead of having two synchronized cameras [21]. Moreover, discoloration of the support due to aging can be present. In order to improve the final result, the state-of-the-art color correction method named Gradient Preserving Spline with Linear Color Propagation (GPS/LCP) presented in [28] is employed to correct the illumination of $I_{2\to 1}$ according to $I_{1}$. Specifically, the color map $g_{\mathrm{GPS}/\mathrm{LCP}}\left(I_{1},I_{2\to 1}\right)=C$, with C:R→R is used to obtain the color corrected image $I_{2\to 1}^{⋆}$ according to

(3) $$I_{2\to 1}^{⋆}\left(x,y\right)=C\left(I_{2\to 1}\left(x,y\right)\right)$$

where, in the error free ideal case, it must hold that $I_{1}=I_{2\to 1}^{⋆}$ (see Figure 2l). The GPS/LCP color correction method is able to preserve the image content and works also in the case of not perfectly aligned images. Color correction decreases the resynthesis error. This can be noted by comparing the error map of $I_{2\to 1}^{⋆}$ (Figure 2m) with the error map of $I_{2\to 1}$ (Figure 2k), see for instance the error corresponding to the dark background above the left table. Clearly, if $I_{2\to 1}$ presents better illumination conditions than $I_{1}$, it is also possible to correct $I_{1}$ according to $I_{2\to 1}$.

### 2.3. Data Fusion

**Figure 4:**
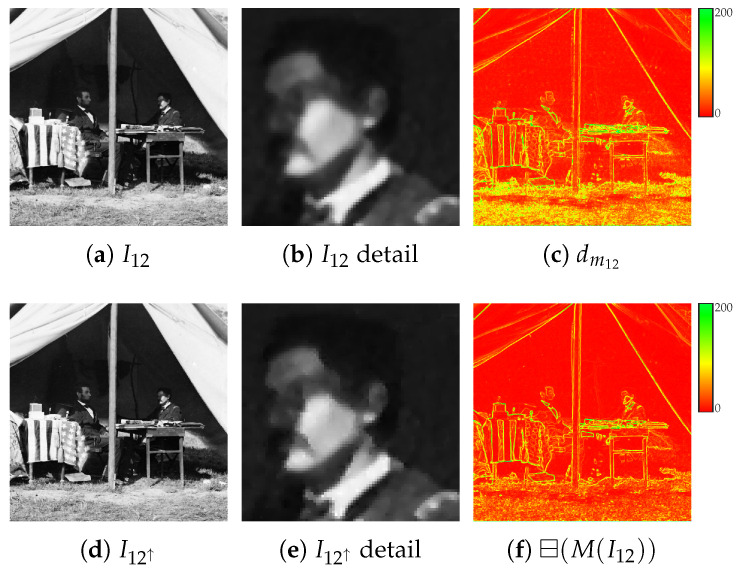
Data fusion step (see Section 2.3): (**a**) stacked median $I_{12}$ obtained from $I_{1}\cup I_{2\to 1}^{⋆}$, (**b**) details of $I_{12}$, (**c**) the stacked median $d_{m_{12}}$ of the gradient magnitudes of $I_{1}$ and $I_{2\to 1}^{⋆}$, (**d**) the gradient-enhanced image $I_{12^{↑}}$, (**e**) a detail of $I_{12^{↑}}$, (**f**) the gradient magnitude $⊟(M\left(I_{12}\right))$ of the stacked median image $I_{12}$. Best viewed in color. The reader is invited to zoom in on the electronic version of the manuscript in order to better appreciate the visual differences.

Given the reference image $I_{1}$ and the synthesized one obtained from the auxiliary view $I_{2\to 1}^{⋆}$ after the illumination post-processing, the two images are blended into a new image $I_{12}$ according to the *stacked median* operator (see Figure 4a)

(4) $$I_{12}=⊟\left(I_{1}\cup I_{2\to 1}^{⋆}\right)$$

**Figure 5:**
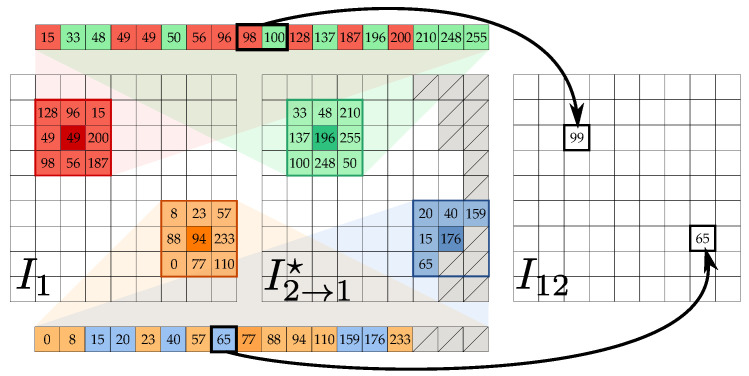
Application of the stacked median operator ⊟ for computing $I_{12}$ from $I_{1}\cup I_{2\to 1}^{⋆}$. At pixel (x,y), the stacked median operator takes the union of the corresponding 3×3 local neighbourhoods for each image of the input set (in the example, the union of the red and green neighbourhoods, and the union of the orange and blue ones, missing data are represented in the figure as gray ticked boxes) and assigns its median intensity value to the point (x,y) in the new image. Best viewed in color.

The stacked median ⊟({I}) for a set of images {I} outputs a new image defined so that image intensity at pixel (x,y) is the median intensity value computed on the union of the pixels in the 3×3 local neighbourhood windows centered at (x,y) on each image of the set (see Figure 5). Notice that the *median stacking* operation typically found as a blending tool in image manipulation software corresponds to the proposed stacked median operator with degenerate 1×1 local windows. Unlike median stacking, the proposed definition does not require more than two input images and considers pixel neighborhoods, i.e., it works locally and not pointwise. Additionally, in case of missing data in $I_{2\to 1}^{⋆}$, the stacked median acts as a standard 3×3 median filter. With this operator, dirt, scratches and other signs of photographic age or damages are effectively removed from $I_{12}$, but high frequency details can be lost in the process, due to the 3×3 filtering (see Figure 4b). These are partially re-introduced by considering a blended version of the gradient magnitude

(5) $$d_{m_{12}}=⊟\left(M\left(I_{1}\right)\cup M\left(I_{2\to 1}^{⋆}\right)\right)$$

(see Figure 4c) obtained as the stacked median of eight possible gradient magnitudes, four for each of the $I_{1}$ and $I_{2\to 1}^{⋆}$ images, to further enhance finer details. Each gradient magnitude image in the set M(I) for a generic image *I* is computed as

(6) $$d_{m}={\left(d_{x}^{2}+d_{y}^{2}\right)}^{\frac{1}{2}}$$

pixelwise, where the image gradient direction pairs $\left(d_{x},d_{y}\right)$ are computed by the convolution of *I* with the following four pairs of kernel filters:

(7) $$\left\{\left(\left[\begin{array}{ccc} 0 & 0 & 0\\ 0 & -1 & 1\\ 0 & 0 & 0 \end{array}\right],\left[\begin{array}{ccc} 0 & 1 & 0\\ 0 & -1 & 0\\ 0 & 0 & 0 \end{array}\right]\right),\;\left(\left[\begin{array}{ccc} 0 & 0 & 1\\ 0 & -1 & 0\\ 0 & 0 & 0 \end{array}\right],\left[\begin{array}{ccc} 1 & 0 & 0\\ 0 & -1 & 0\\ 0 & 0 & 0 \end{array}\right]\right),\;\left(\left[\begin{array}{ccc} 0 & 0 & 0\\ 1 & -1 & 0\\ 0 & 0 & 0 \end{array}\right],\left[\begin{array}{ccc} 0 & 0 & 0\\ 0 & -1 & 0\\ 0 & 1 & 0 \end{array}\right]\right),\;\left(\left[\begin{array}{ccc} 0 & 0 & 0\\ 0 & -1 & 0\\ 1 & 0 & 0 \end{array}\right],\left[\begin{array}{ccc} 0 & 0 & 0\\ 0 & -1 & 0\\ 0 & 0 & 1 \end{array}\right]\right)\right\}$$

Notice that $d_{m_{12}}\neq ⊟\left(M\left(I_{12}\right)\right)$ in the general case (compare Figure 4c with Figure 4f). Consider for now only a single derivative pair $\left(d_{x},d_{y}\right)$ of $I_{12}$: Each pixel intensity $I_{12}\left(x,y\right)$ is incremented by a value v(x,y) satisfying

(8) $${\left(d_{x}+\frac{v}{2}\right)}^{2}+{\left(d_{y}+\frac{v}{2}\right)}^{2}=\frac{d_{m}^{2}-d_{m_{12}}^{2}}{2}$$

This equation has a twofold solution

(9) $$v^{⋆}=\pm {\left(2d_{x}d_{y}-d_{m_{12}}^{2}\right)}^{\frac{1}{2}}-\left(d_{x}+d_{y}\right)$$

In the case of two real $v^{⋆}$ solutions, *v* is chosen as $v\left(x,y\right)=\arg\min_{\bar{v}\in v^{⋆}}\left|\bar{v}\right|$ in order to alter $I_{12}$ as little as possible. In the case of complex solutions, v(x,y) is set to 0. The final gradient-enhanced image is then obtained as

(10) $$I_{12^{↑}}=I_{12}+v$$

(see Figure 4d,e for details). Since four different *v* values are obtained for each of the four derivative pairs of Equation (7), their average value is actually employed.

### 2.4. Refinement

**Figure 6:**
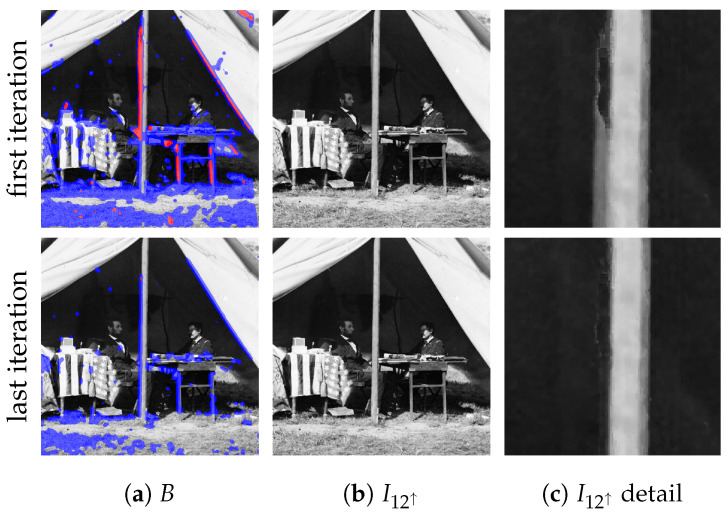
Refinement step (see Section 2.4). First (top row) and last (bottom row) iterations of the detection and adjustment sub-steps. (**a**) detection mask *B* at the beginning of the iteration, (**b**) updated $I_{12^{↑}}$ at the end of the iteration and (**c**) details of (**b**). Pixels to be adjusted using $L^{\prime }$ (*L*) are underlined in the images by saturating the red (blue) channel. By inspecting the details, it can be seen that the ghosting effect is removed. Best viewed in color. The reader is invited to zoom in on the electronic version of the manuscript in order to better appreciate the visual differences.

As already noted, the optical flow may be not perfect, causing the presence of wrong data in the image synthesis and hence in the data fusion process described in the previous step. To alleviate this issue, an iterative error-driven image correction step is introduced, where each iteration can be split into two sub-steps:

1. Detection. A binary correction mask is computed by considering the error image $E={\left(I_{1}-I_{12^{↑}}\right)}^{2}$ the 11×11 local window L(x,y) centered at each (x,y). Given $L^{\prime }\left(x,y\right)\subseteq L\left(x,y\right)$ as the subset of pixels with intensity values lower than the 66% percentile on L(x,y), the pixel (x,y) is marked as requiring adjustment if the square root of the average intensity value on $L^{\prime }\left(x,y\right)$ is higher than t=16 (chosen experimentally). This results in a binary correction mask *B* that is smoothed with a Gaussian kernel and then binarized again by a threshold value of 0.5. As clear from Figure 6a, using the percentile-based subset $L^{\prime }\left(x,y\right)$ is more robust than working with the whole window L(x,y).
2. Adjustment. Data fusion is repeated again after updating pixels on $I_{2\to 1}^{⋆}$ that need to be adjusted with the corresponding ones of $I_{12^{↑}}$. Since $I_{12^{↑}}$ is a sort of average between $I_{1}$ and $I_{2\to 1}^{⋆}$, the operation just described pushes marked pixels towards $I_{1}$. At the end of this step, the gradient enhanced image $I_{12^{↑}}$ is also updated accordingly and, in case of no further iterations, it constitutes the final output.

Iterations stop when no more pixels to be adjusted are detected in the updated $I_{12^{↑}}$ or when the maximum number of iterations is reached (see Figure 6). A maximum of four iterations is carried out, since it was verified experimentally that data fusion typically converges to $I_{1}$ within this number of steps.

### 2.5. Guided Supersampling

**Figure 7:**
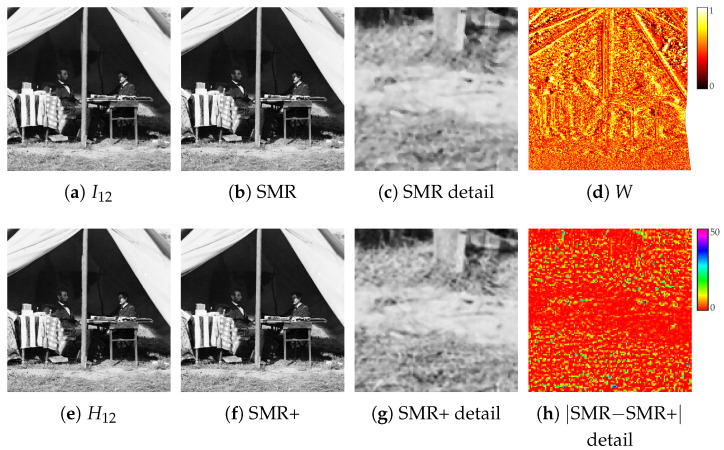
Guided supersampling step (see Section 2.5): (**a**) SMR stacked median $I_{12}$, (**b**) final restored image and (**c**) details of it, (**d**) weight mask *W* for the guided supersampling, (**e**) SMR+ weighted stacked median $H_{12}$, (**f**) final restored image, (**g**) a detail of it, and (**h**) its differences with respect to the SMR output. Best viewed in color. The reader is invited to zoom in on the electronic version of the manuscript in order to better appreciate the visual differences.

Previous steps describe the original SMR implementation [23]. In order to preserve more fine details of the input images, a better image fusion is proposed hereafter, where the original coarse blended image $I_{12}$ (Equation (4)) is employed to guide a refinement on the basis of supersampling (see Figure 7).

Let $W_{1}$ denote the image obtained by averaging $|I_{1}-I_{12}|$ on a 3×3 window, and similarly $W_{2}$ the one obtained with $|I_{2}-I_{12}|$. The weight mask *W* is computed as $W_{1}/\left(W_{1}+W_{2}\right)$ pixelwise, followed by the convolution with a Gaussian with a standard deviation of four pixels (see Figure 7d). A value of *W* close to 0 (1) for a given pixel implies that the local neighborhood of that pixel in $I_{1}$ ($I_{2}$) is very likely less noisy and more artefact-free than $I_{2}$ ($I_{1}$). The mask *W* is used to define a *weighted stacked median*

(11) $$H_{12}={⊟}_{W}\left(I_{1}^{{}_{\times 2}},I_{2\to 1}^{{⋆}_{\times 2}}\right)$$

where the superscript ×2 indicates the bicubic rescaling by a factor two for supersampling (see Figure 7e). Explicitly, the weighted stacked median at (x,y) is obtained as the median of the intensities of $V_{1}\left(x,y\right)\cup V_{2}\left(x,y\right)$, where $V_{1}\left(x,y\right)\subseteq I_{1}$ is the subset of the pixels in the 3×3 local neighbourhood of (x,y) containing the $\left\lfloor w\times \min\left(1-W\left(x,y\right),w^{\prime }\right)\right\rfloor$ intensity values closest to $I_{12}^{{}_{\times 2}}\left(x,y\right)$, with $w=3^{2}\times 2$ and $w^{\prime }=\left(3^{2}+1\right)/\left(2\times 3^{2}+1\right)$, and likewise for $V_{2}\left(x,y\right)\subseteq I_{2}$ containing the pixels with the $\left\lfloor w\times \min\left(W\left(x,y\right),w^{\prime }\right)\right\rfloor$ closest values. In other words, the number of considered samples for the median taken from each image is proportional to the weight W(x,y). The cardinalities of the subsets $V_{1}$ and $V_{2}$ for the different weight ranges are explicitly shown in Table 1.

The high resolution blended image $H_{12}$ replaces $I_{12}$ in the next steps of the method (see Section 2.3 and Section 2.4), $I_{1}$ and $I_{2}$ being also replaced accordingly by $I_{1}^{{}_{\times 2}}$ and $I_{2}^{{}_{\times 2}}$. The final output is scaled down to the original input size. With respect to the original SMR implementation, the use of guided supersampling in SMR+ preserves better fine details, also improving further the restoration process (compare Figure 7c,g). Notice that, after each refinement sub-step (see Section 2.4), the coarse $I_{12}$ image needed to guide the process is generated by the stacked median between $I_{1}$ and $I_{12^{↑}}^{{}_{\times 2}}$ scaled down to the original size.

## 3. Evaluation

### 3.1. Dataset

**Figure 8:**
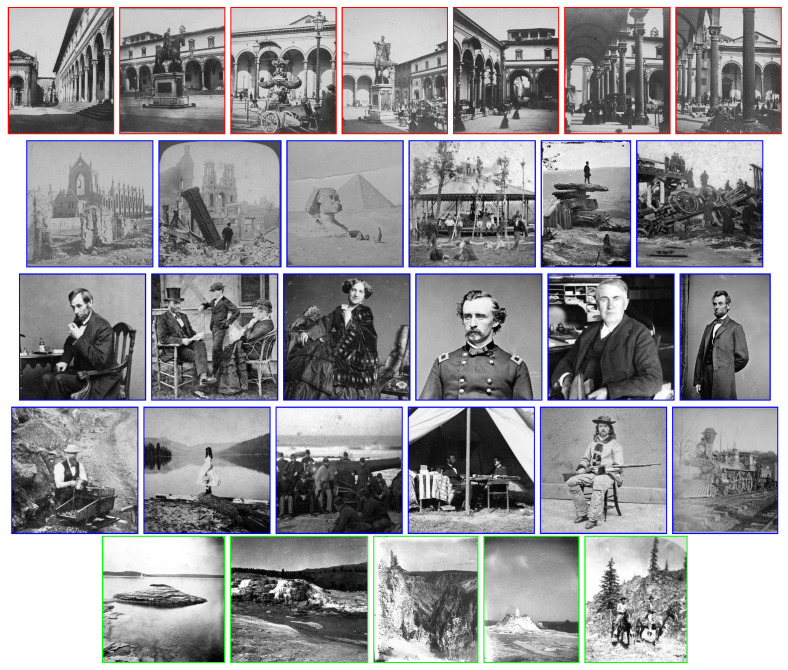
Left frames for some historical stereo pairs. Image frames for Hautmann’s, Stereoscopic Photos and USGS datasets are framed, respectively, in red, blue and green. Best viewed in color and zoomed in with the electronic version of the manuscript.

In order to evaluate the proposed approach, we built a new dataset including historical stereo pairs from different sources. The left frames of the selected stereo pairs are shown as reference in Figure 8.

A first set of seven stereo pairs belongs to the collection of stereograms by Anton Hautmann, one of the most active photographers in Florence between 1858 and 1862. Part of Hautmann’s collection is described in [21]. The seven stereo pairs used in this work depict different viewpoints of Piazza Santissima Annunziata in Florence as it was in the middle of the 19th century. Inspecting these photos (see Figure 8, red frames), it can be noticed that the image quality is very poor. In particular, the pairs are quite noisy, with low definition and contrast, include saturated or blurred areas and also show scratches and stains.

A second set includes 35 stereo pairs and increases the original set of ten images employed in [23]. These stereo pairs have been gathered from the Stereoscopic History Instagram account (https://www.instagram.com/stereoscopichistory/, accessed on 1 April 2021, see Figure 8, blue frames for some examples) and contain landscape pictures of urban and natural scenes as well as individual or group portraits. This set is the most challenging one, since its images are heavily corrupted by noise and other artefacts.

A third set of five images was collected from the U.S. Geological Survey (USGS) Historical Stereoscopic Photos account on Flickr (https://www.flickr.com/photos/usgeologicalsurvey/, accessed on 1 April 2021), and represents natural landscapes (see Figure 8, green frames), except for the last one which also includes two horsemen with their mounts. The quality of these images is similar to that of the first set, but strong vignetting effects are also present.

### 3.2. Compared Methods

The proposed SMR and SMR+ are compared against Block Matching 3D (BM3D) [7], Deep Image Prior (DIP) [13] and the recent BOPBtL [26]. BM3D and DIP are, respectively, a handcrafted and deep generic denoising methods, while BOPBtL is a deep network specifically designed for old photo restoration. These methods currently represent the state-of-the-art in this research area.

For BM3D, the legacy version was employed, since, according to our preliminary experiments, the new version including correlated noise suppression did not work well for our kind of images. The BM3D σ parameter, the only one present, was set to 7 and 14, values that, according to our experiments, gave the best visual results. In particular, σ=14 seems to work better than σ=7 in the case of higher resolution images. Besides applying the standard BM3D on the reference image, a modified version of this method was implemented in order for BM3D to benefit from the stereo auxiliary data. Since BM3D exploits image self-correlation to suppress noise, the modified BM3D generates auxiliary sub-images by placing side by side two corresponding 96×96 patches from $I_{1}$ and $I_{2\to 1}^{⋆}$, then runs the original BM3D on each sub-image and finally generates the output by collecting the blocks from each sub-image corresponding to the 32×32 central $I_{1}$ patches. No difference in the results with respect to the standard BM3D was observed, which plausibly implies that corresponding patches for $I_{1}$ and $I_{2\to 1}^{⋆}$ are not judged as similar to each other by BM3D.

In the case of DIP, the borders of the input images were cropped due to network architectural constraints: These missing parts were replaced with content from the original input images.

**Figure 9:**
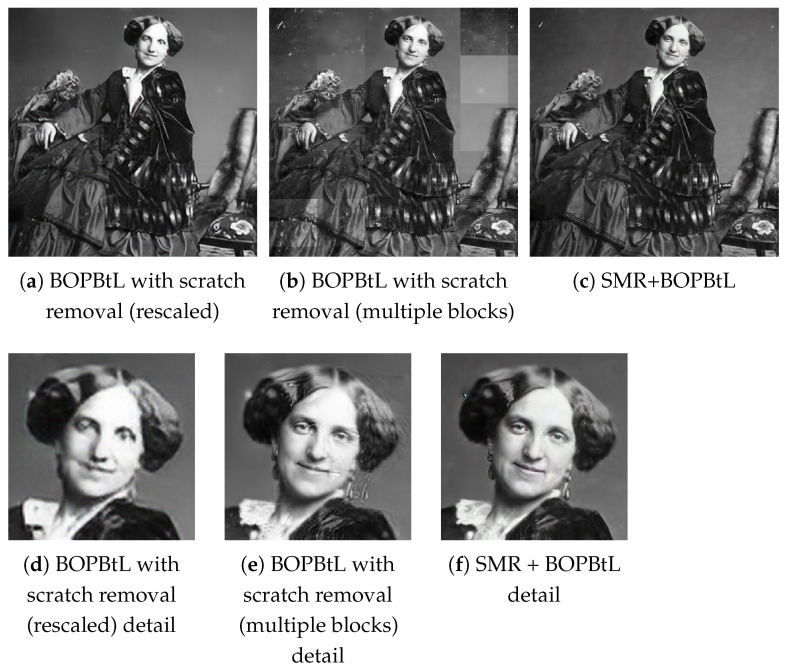

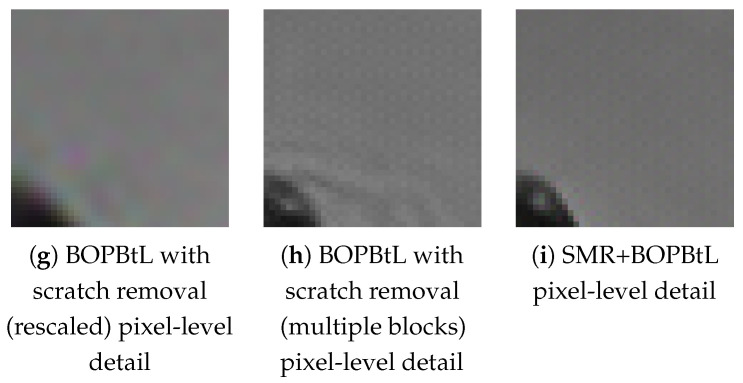
Results of BOPBtL with scratch removal or in combination with SMR+ on the same stereo pair of Figure 1. Notice that the visual pleasant results of (**a**) are due to the frequency cutoff caused by rescaling and disappear at a larger viewing scale such in (**d**). Best viewed in color. The reader is invited to zoom in on the electronic version of the manuscript in order to better appreciate the visual differences.

Concerning BOPBtL, the scratch removal option was disabled since it caused the network to crash. This is a known issue related to the high memory requirement exceeding the standard 12 GB GPU amount to run the network on standard image input (https://github.com/microsoft/Bringing-Old-Photos-Back-to-Life/issues/, accessed on 1 April 2021), and does not occur only when the input image size is small. To circumvent this problem, two solutions were attempted, yet without satisfying results. Specifically, in the first solution, the input image was rescaled to a fixed size (from 50% to 33% of the its original size), but the final result lost too many details (see Figure 9a). In the second solution, the input was processed in separated blocks, causing a lack of global consistency in the output (see Figure 9b). Moreover, in both solutions, the chessboard artefact effect, typical of many deep networks that resynthesize images, looked more evident than in the original BOPBtL implementation. BOPBtL was employed to post-process the output of SMR+, which was denoted as SMR+BOPBtL in the results (see Figure 9c).

### 3.3. Results

**Figure 10:**
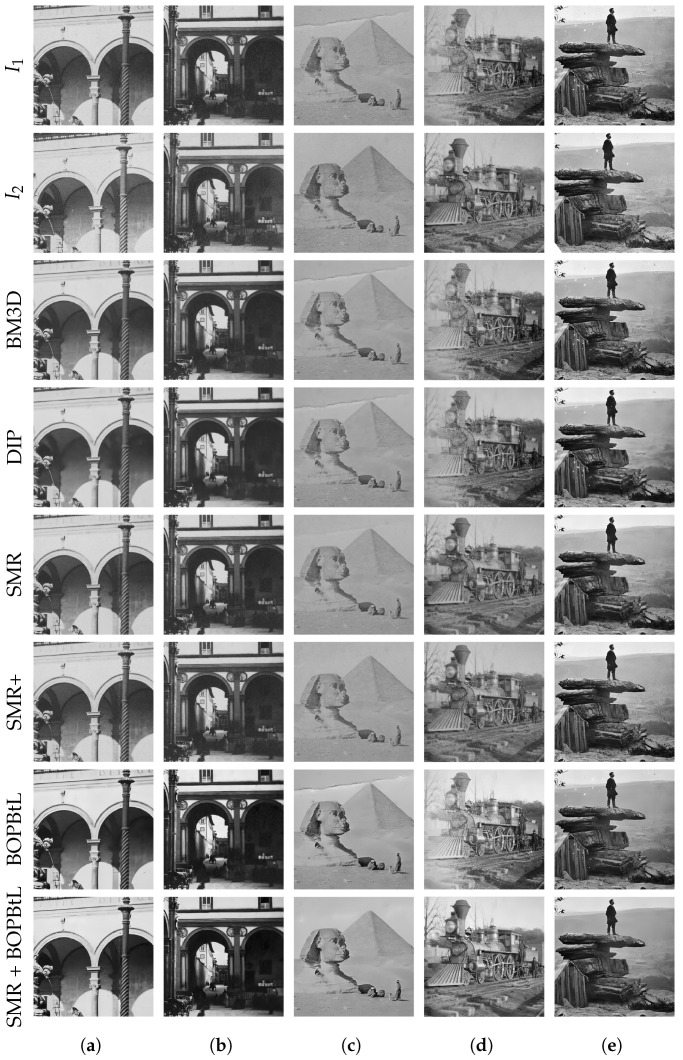
Qualitative visual comparison of the methods under test. Best viewed in color. The reader is invited to zoom in on the electronic version of the manuscript in order to better appreciate the differences.

**Figure 11:**
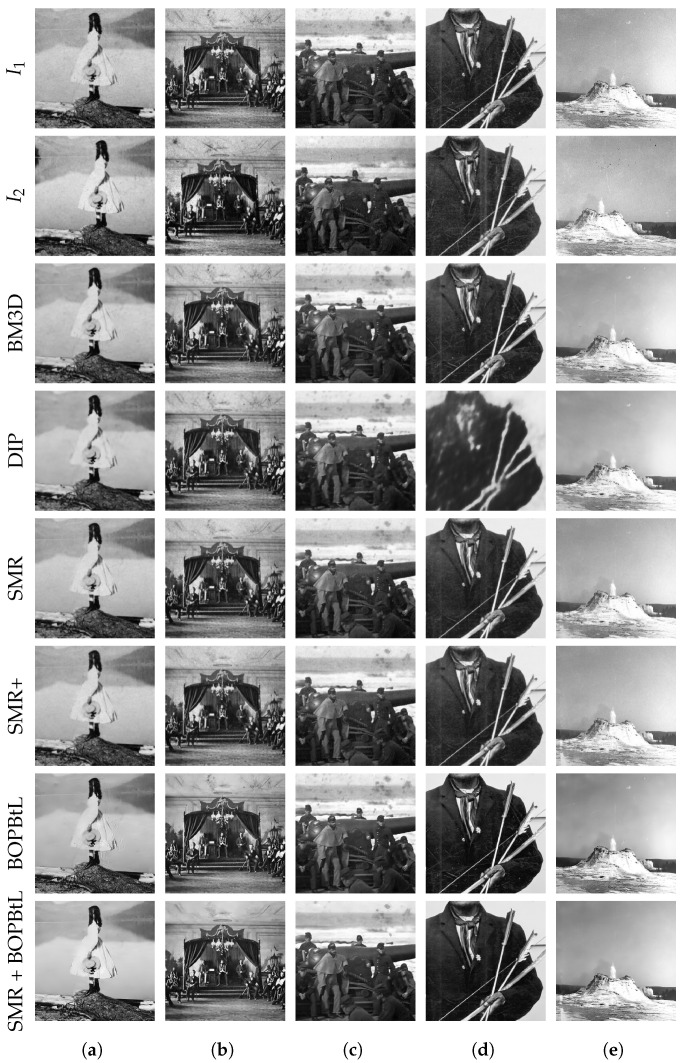
Qualitative visual comparison of the methods under test. Best viewed in color. The reader is invited to zoom in on the electronic version of the manuscript in order to better appreciate the differences.

Figure 10 and Figure 11 show some examples of the results obtained with the compared methods. For a thorough visual qualitative evaluation, the reader is invited to inspect the full-resolution results obtained on the whole dataset, which are included in the additional material (https://drive.google.com/drive/folders/1Fmsm50bMMDSd0z4JXOhCZ3hPDIXdwMUL). From a direct visual inspection of the results, BM3D and DIP often seem to oversmooth relevant details in the image, with BM3D producing somewhat better results than DIP, which sometimes simply fails to obtain a reasonable output (see Figure 11d, row DIP). BOPBtL is able to bring out fine details, providing altogether a locally adaptive smoothing and contrast enhancement of the image, with satisfactory results. Nevertheless, none of the previous methods is able to detect and compensate for dust, scratches and other kinds of artefacts that conversely may even be amplified in the restoration process, as one can check by locating dust spots and sketches in Figure 10e, rows BM3D, DIP and BOPBtL. This problem is mostly evident for BOPBtL, where image artefacts are heavily boosted together with finer details.

Conversely, SMR-based methods are able to solve these issues by exploiting the additional information present in the auxiliary image, with the exception of very severe conditions such as the stains appearing in the right skyline of Figure 11c, for which, anyways, SMR-based methods still get the best restoration of all. SMR-based methods also successfully enhance the image contrast, as it happens for the window in the dark spot under the right arcade in Figure 10b, rows SMR and SMR+. When image degradations are even more severe than that, good results can nevertheless be obtained by forcing the illumination of the auxiliary image into the reference (see Section 2.2), as done for Figure 10d, rows SMR, SMR+ and SMR+BOPBtL. Concerning the guided supersampling introduced for SMR+, this is able not only to preserve high frequency details (see again Figure 7), but also to better clean-up the image, as one can notice by inspecting the removed scratch from Figure 10c, row SMR+. Guided supersampling also alleviates spurious artefacts arising from inaccurate optical flow estimation as in the case of the light pole of Figure 10a (compare rows SMR and SMR+). Only in few cases of very inaccurate optical flow estimation is SMR+ unable to fix inconsistencies and generates some spurious artefacts as in the bottom-left white scratch in Figure 11e, rows SMR+ and SMR + BOPBtL. Finally, it can be noted that SMR + BOPBtL is able to take the best from both the methods, i.e., the artefact removal from SMR+ and the image enhancement from BOPBtL, and provides very visually striking results.

Table 2 reports the score obtained by the compared methods on the images discussed so far according to popular no-reference quality assessment metrics. Specifically, scores are reported for the Blind/Referenceless Image Spatial Quality Evaluator (BRISQUE) [29], the Naturalness Image Quality Evaluator (NIQE) [30] and Perception based Image Quality Evaluator (PIQE) [31]. Due to the lack of ground-truth clean data and of a well-defined image model for the generation of synthetic images with the same characteristics of the input image under evaluation, image quality measurements requiring a reference image such as the Structural Similarity Index (SSIM) [32] cannot be applied. By inspection of the scores obtained, it clearly emerges that these quality metrics do not reflect the human visual judgment, hence they are unsuitable for a reliable quantitative evaluation in this specific application scenario. In particular, there is no agreement among the various metrics and, in about half of the cases, the input image even gets a better score than the restored one, in contrast with the human visual assessment. Furthermore, SMR+ and SMR + BOPBtL obtain worse scores than the original images or BOPBtL in the cases where SMR-methods successfully cleaned the image by removing strong image artefacts, again in contrast with human judgment (see Figure 11b,d). A possible explanation of this behavior is that these metrics only rely on low-level, local image properties and not on high-level, semantic image characteristics. Hence, they are unable to distinguish between fine image details and artefacts. Nevertheless, according to Table 2, SMR+, with or without BOPBtL, shows good results under these blind quality assessment metrics, implying that it is able not only to remove structural artefacts from the original image, but also to maintain high quality visual details besides the semantic interpretation of the scene.

Concerning running times, BM3D, BOPBtL and DIP require respectively 10 s, 30 s and 20 min on average for processing the dataset images, while SMR and SMR+ take respectively 6 min and 9 min. The running environment is a Ubuntu 20.04 system running on an Intel Core i7-3770K with 8 GB of RAM equipped with a 12 GB NVIDIA Titan XP. BM3D is a Matlab optimized .mex file, BOPBtL and DIP implementations run on Pytorch exploiting GPU acceleration, while, with the exception of RAFT optical flow estimation, SMR and SMR+ are based on non-optimized Matlab code running on CPU. For both SMR and SMR+ the times include the image resynthesis and color correction steps that take 4.5 min altogether on average. Under these considerations, both SMR and SMR+ running times are reasonable for offline applications. None of the compared methods can be used for real-time applications, as in the best case corresponding to BM3D, 10 s are required for processing the input image.

## 4. Conclusions and Future Work

This paper proposed a novel method for the fully automatic restoration of historical stereo photographs. By exploiting optical flow, the auxiliary view of the stereo frame is geometrically and photometrically registered onto the reference view. Restoration is then carried out by fusing the data from both images according to our stacked median approach followed by gradient adjustments aimed at preserving details. Guided supersampling is also introduced and successfully applied for enhancing finer details and simultaneously providing a more effective artefact removal. Finally, an iterative refinement step driven by a visual consistency check is performed in order to remove the artefacts due to optical flow estimation errors in the initial phase.

Results on several historical stereo pairs show the effectiveness of the proposed approach that is able to remove most of the image defects including dust and scratches, without excessive smoothing of the image content. The approach works better than its single-image denoising competitors, thanks to the ability of exploiting stereo information. As a matter of fact, single-image methods have severe limitations in handling damaged areas, and usually produce more blurry results. Nevertheless, experimental results show that single image BOPBtL, when cascaded with our approach into SMR + BOPBtL, can achieve remarkably good performances.

Future work will investigate novel solutions to refine the optical flow in order to reduce pixel mismatches. A further research direction will be towards consolidating the stacked median approach as an image blending technique. Finally, the proposed method will be extended and adapted to the digital restoration of historical films.

## Figures and Tables

**Table 1:** The cardinality of the sets $V_{1}\left(x,y\right)$ and $V_{2}\left(x,y\right)$ according to the weight W(x,y) range (see Section 2.5).

infW(x,y)	0.00	0.05	0.11	0.16	0.21	0.26	0.32	0.37	0.42	0.47	0.53	0.58	0.63	0.68	0.74	0.79	0.84	0.89	0.95
supW(x,y)	0.05	0.11	0.16	0.21	0.26	0.32	0.37	0.42	0.47	0.53	0.58	0.63	0.68	0.74	0.79	0.84	0.89	0.95	1.00
$|V_{1}\left(x,y\right)|$	9	9	9	9	9	9	9	9	9	9	8	7	6	5	4	3	2	1	0
$|V_{2}\left(x,y\right)|$	0	1	2	3	4	5	6	7	8	9	9	9	9	9	9	9	9	9	9

**Table 2:** No-reference assessment metric results (lower values are better). Values in bold indicate the best score among the compared methods. Scores that are better in the original images than in the restored ones are underlined.

		$I_{1}$	BM3D	DIP	SMR	SMR+	BOPBtL	SMR+ BOPBtL
Figure 1 and Figure 9	BRISQUE	41.89	54.34	51.47	53.11	43.46	**24.15**	24.20
	NIQE	4.23	5.31	5.31	5.09	**3.98**	4.09	4.24
	PIQE	45.97	78.93	85.33	50.60	46.35	**22.55**	25.90
Figure 10a	BRISQUE	10.74	46.03	31.11	42.18	33.06	**25.41**	31.37
	NIQE	2.79	3.83	3.94	**3.28**	3.76	4.05	4.08
	PIQE	25.02	79.24	81.50	43.32	**28.09**	38.50	35.35
Figure 10b	BRISQUE	9.84	48.68	35.95	41.57	29.69	**14.17**	34.69
	NIQE	3.16	4.07	3.92	**2.92**	3.34	3.65	4.01
	PIQE	29.73	78.53	78.16	37.26	**23.61**	29.98	34.31
Figure 10c	BRISQUE	9.26	44.97	31.28	38.29	33.94	**12.13**	19.06
	NIQE	2.79	4.22	4.11	**3.47**	4.04	5.43	5.31
	PIQE	15.80	60.33	53.28	42.81	23.02	20.30	**20.00**
Figure 10d	BRISQUE	14.57	31.93	22.82	36.91	25.66	15.89	**10.96**
	NIQE	2.61	**3.11**	3.72	3.49	3.65	3.97	3.62
	PIQE	9.31	43.23	52.66	38.28	24.24	**10.48**	11.76
Figure 10e	BRISQUE	12.85	30.58	28.31	31.95	**22.40**	29.13	28.87
	NIQE	2.17	2.26	3.30	3.13	**2.92**	4.05	3.97
	PIQE	27.52	42.54	45.40	40.00	24.43	**14.67**	16.92
Figure 11a	BRISQUE	42.58	48.03	40.26	51.88	41.23	38.48	**39.21**
	NIQE	3.80	4.77	4.97	4.66	**3.93**	4.57	4.75
	PIQE	26.39	74.37	79.44	45.89	36.91	**13.28**	14.60
Figure 11b	BRISQE	39.15	49.22	53.80	45.41	40.85	**14.75**	17.74
	NIQE	4.33	5.43	5.78	4.93	**4.15**	4.32	4.56
	PIQE	28.96	82.41	84.95	46.49	38.68	**15.54**	17.70
Figure 11c	BRISQE	30.43	52.90	55.07	52.86	39.59	25.54	**20.06**
	NIQE	3.13	5.22	5.53	4.25	**3.20**	4.59	4.36
	PIQE	17.20	85.95	88.53	43.98	30.33	**25.39**	27.83
Figure 11d	BRISQUE	28.40	45.63	47.19	41.24	31.51	**22.09**	23.47
	NIQE	2.11	4.17	6.28	3.89	**2.85**	3.49	3.85
	PIQE	31.65	72.88	94.84	48.02	36.64	**20.68**	22.81
Figure 11e	BRISQUE	40.12	38.54	37.95	**20.01**	22.15	38.12	22.07
	NIQE	6.27	3.49	4.08	2.84	**3.06**	4.60	4.42
	PIQE	58.45	51.79	48.00	19.77	**13.28**	13.35	11.45

## Data Availability

Additional material including code, dataset and evaluation results are freely available online at https://drive.google.com/drive/folders/1Fmsm50bMMDSd0z4JXOhCZ3hPDIXdwMUL.
